# A Perturb-seq map of a differentiation hub reveals synergistic vulnerabilities in KMT2A-rearranged acute myeloid leukemia

**DOI:** 10.1038/s41375-026-02917-2

**Published:** 2026-03-25

**Authors:** Sajesan Aryal, Brittany M. Curtiss, Xinyue Zhou, Rui Lu, Changde Cheng

**Affiliations:** 1 Department of Medicine, Division of Hematology/Oncology, University of Alabama at Birmingham, Birmingham, AL USA; 2 O’Neal Comprehensive Cancer Center, University of Alabama at Birmingham, Birmingham, AL USA; 3 Medical Scientist Training Program (MSTP), University of Alabama at Birmingham, Birmingham, AL USA; 4 Department of Biomedical Informatics and Data Science, University of Alabama at Birmingham, Birmingham, AL USA; 5 Institute for Cancer Outcomes and Survivorship, University of Alabama at Birmingham, Birmingham, AL USA

**Keywords:** Cancer genomics, Cancer

## Abstract

KMT2A-rearranged acute myeloid leukemia is driven by epigenetic dependencies yet remains clinically resistant to therapies targeting individual regulators, indicating that resistance reflects compensatory regulation across an epigenetic network. A systematic understanding of this compensatory network has been lacking. To address this gap, we utilized Perturb-seq screening to systematically map the functional architecture of this network. We uncovered a compensatory epigenetic circuit, where a synergistic hub including KAT6A, Menin, and DOT1L converges to silence a core differentiation program (the ‘Myeloid Program’), thereby maintaining leukemic identity. The activity of this program strongly correlated with favorable survival in large patient cohorts. While individual perturbations of hub components only partially derepress this program, their simultaneous pharmacological inhibition collapses the circuit’s buffering capacity, leading to robust reactivation of the Myeloid Program and potent synergistic anti-leukemic activity. Our model also shows that disruption of antagonistic regulators of the Myeloid Program, such as the PRC1.1 component *PCGF1*, confers strong resistance to DOT1L inhibition. Finally, the Myeloid Program is a predictive biomarker, where high baseline activity defined a vulnerable state that could be selectively targeted by MEK, AKT, and mTOR inhibitors. Together, these findings establish a framework for identifying circuit-level epigenetic compensation and for rationally designing precision combination therapies that restore differentiation or target state-dependent vulnerabilities in acute myeloid leukemia.

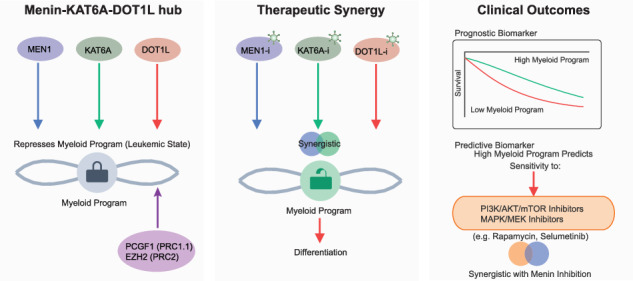

## Introduction

KMT2A-rearranged acute myeloid leukemia (KMT2A-r AML) is an aggressive subtype marked by a differentiation block and poor clinical outcomes [1]. Although targeted therapies against individual epigenetic regulators, such as Menin or DOT1L, have shown initial activity, responses are often incomplete and short-lived [2–4]. This suggests that therapeutic resistance is not simply due to partial target engagement but reflects an underlying regulatory architecture capable of buffering against single perturbations. Yet the structure of this epigenetic network and the principles by which it maintains leukemic identity remain undefined. Whether resistance arises from compensatory interactions among chromatin-modifying complexes and how these interactions converge on cell fate programs is unknown. Addressing this gap is critical for designing rational, durable combination strategies in AML.

The KMT2A-r AML phenotype is epigenetically governed by two distinct, yet functionally convergent, classes of regulators. Activating complexes, such as Menin-KMT2A fusions in cooperation with histone acetyltransferases (HATs) like KAT6A, drive oncogenic gene expression programs (e.g., HOX genes) to promote self-renewal [2, 5, 6]. Concurrently, Polycomb repressive complexes (PRC1/PRC2) silence critical differentiation genes (e.g., CEBPA), thereby locking cells in an immature state [7]. The central challenge, however, is that the relationship between these activating and repressive forces is not a simple antagonism; it is highly context-dependent and poorly understood. For instance, Menin-KMT2A and Polycomb complexes directly oppose each other at bivalent gene promoters [8], yet inhibition of Menin can be rendered ineffective by compensatory Polycomb-mediated repression at other noncanonical genomic loci [9]. Furthermore, recent studies have demonstrated that genetic dependency on individual nodes such as KAT6A does not reliably translate into robust single-agent pharmacological efficacy, further underscoring the presence of functional redundancy within the broader epigenetic network [10]. This incomplete understanding presents a fundamental barrier to designing rational combination therapies and highlights the need for systematic approaches.

Recent advances in single-cell CRISPR screening (Perturb-seq), for instance, now enable the systematic dissection of gene networks [11], presenting a novel opportunity to resolve the problem of epigenetic cooperativity in KMT2A-r AML. Here, we use Perturb-seq to systematically resolve these compensatory interactions at single-cell resolution, define the higher-order circuit that sustains the leukemic state, and translate this map into precision combination therapies. Specifically, we employed Perturb-seq with computational modeling to comprehensively map the transcriptional impact of perturbing 16 key epigenetic regulators in a KMT2A-r AML model. The objective of this approach was threefold: first, to identify the core gene expression programs critical for maintaining the leukemic state; second, to define the synergistic and antagonistic relationships between regulators that control these programs; and third, to leverage this functional map to discover and validate rational combination therapies. Taken together, our findings provide a new mechanistic model that explains how compensatory epigenetic regulation sustains the leukemic state.

## Results

### High-throughput single-cell perturbation of epigenetic regulators in AML

**Fig. 1:**
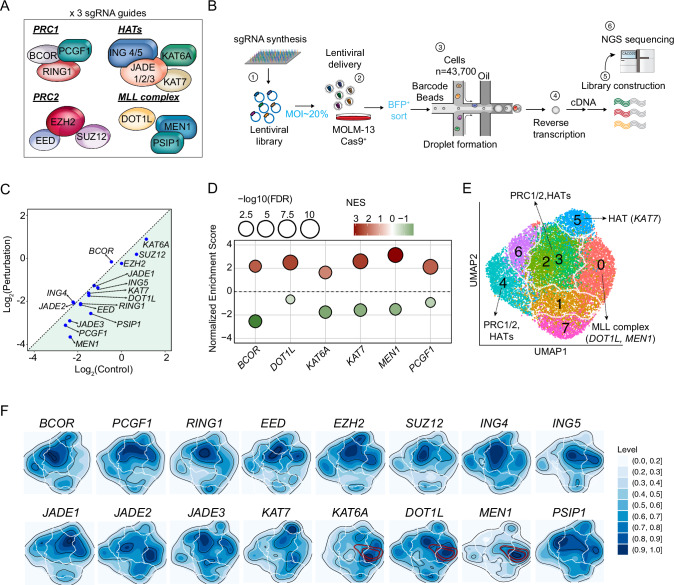
High-throughput single-cell perturbation of epigenetic regulators in AML. **A** Schematic representing the 16 key genes selected for perturbation, grouped by their respective protein complexes: PRC1, PRC2, HATs, and the Menin-MLL complex. The label “x 3 sgRNA guides” indicates that the library of three independent single-guide RNAs was designed and synthesized for each target gene. **B** Schematic of the Perturb-seq experimental workflow. **C** Scatter plot of Log₂(Control) versus Log₂(Perturbation), showing the effect of gene knockout. **D** Bubble plot showing the Normalized Enrichment Score (NES) (y-axis) for each target gene when compared to respective bulk RNA-seq. Bubble size represents statistical significance (−log _10_FDR), and bubble color indicates the NES (red for positive enrichment/upregulation, green for negative enrichment/downregulation). **E** UMAP plot of single-cell transcriptomes. Individual cells are colored by eight clusters (0–7) identified through unsupervised clustering. **F** Density plots showing the spatial expression patterns of key target genes across the UMAP space. The color scale (right) indicates relative cell density.

To systematically investigate the functional roles of Menin-MLL (e.g., *MEN1, DOT1L*), Polycomb repressive complexes (PRC1/PRC2, e.g., *EZH2, SUZ12, PCGF1*), and histone acetyltransferase (HAT) complexes (e.g., *KAT6A, KAT7*) in KMT2A-r AML, we performed Perturb-seq targeting 16 key epigenetic regulators in the MOLM-13 cell line, a typical KMT2A-r AML line with MLL-AF9 gene rearrangement (Fig. 1A, B, Supplementary Table 1). These regulators were selected based on their established roles in chromatin modification and transcriptional regulation, which are critical for KMT2A-r pathogenesis [12–14]. The 16 chromatin regulators were not chosen to represent an unbiased or genome-wide survey of epigenetic dependencies in KMT2A-r AML. Rather, we selected a focused, mechanistically anchored set of upstream, non-redundant nodes from the three core complexes known to sustain the KMT2A-rearranged leukemic state. These factors were prioritized because they represent the minimal set of master regulators whose perturbation reliably produces large, interpretable shifts in leukemic cell identity, enabling inference of higher-order circuit architecture from single-gene knockouts.

Single-cell RNA yielded 31,015 high-quality transcriptomes after quality control filtering. Guide RNA distribution analysis confirmed that 68% of the cells contained a single sgRNA, enabling precise inference of transcriptional effects (Supplementary Fig. 1A). A median of 548 cells per perturbed gene were captured, with 182 cells per guide median (Supplementary Fig. 1B, C). We next confirmed the efficacy of our perturbations by observing significant downregulation of key genes (Fig. 1C). Notably, while most targets were strongly downregulated, some targets, including *BCOR*, did not show the expected downregulation at the transcript level (Fig. 1C). This is likely because CRISPR-induced mutations can lead to alternative mRNA processing events, such as exon skipping or internal ribosomal entry, which allow transcripts to evade nonsense-mediated decay (NMD), resulting in stable mRNA levels despite functional gene disruption [15, 16]. Additionally, the position of the mutation relative to exon-exon junctions can also influence NMD efficiency [17].

Therefore, to validate the functional consequences of our perturbations, we compared our single-cell signatures against corresponding bulk RNA-seq datasets following individual knockout via gene set enrichment analysis (GSEA). For each perturbation, gene sets were defined using the differentially expressed genes identified in the perturb-seq data, and their enrichment was evaluated in the corresponding bulk RNA-Seq data. The GSEA results revealed a high degree of agreement, with significant enrichment of both upregulated and downregulated gene sets in the expected directions (Fig. 1D, Supplementary Table 2). Collectively, these results confirm that genes perturbed in our Perturb-seq data are similarly perturbed in bulk RNA-Seq data, reinforcing the reliability of the perturbation effects observed (Fig. 1C, D). Taken together, these validation steps, particularly the confirmation of the *BCOR* signature, demonstrate that our Perturb-seq platform fully captures the functional impact of epigenetic perturbations, providing a robust foundation for studying novel genetic interactions.

To dissect the functional landscape of epigenetic regulation, we performed an unsupervised projection of single-cell transcriptomes via UMAP. This analysis revealed that perturbations segregated according to their functional protein complex (Fig. 1E). The MLL and KAT7 complexes induced well-defined transcriptional states, which localized to distinct regions of the UMAP. Conversely, perturbations of PRC1/2 and other HAT complexes formed a connected continuum, highlighting a more heterogeneous state of transcriptional consequences (Fig. 1E, F, Supplementary Fig. 1F). This highlighted the varying magnitude of their transcriptional impact. To quantify this, we analyzed the number of differentially expressed (DE) genes for each perturbation. The perturbations of the Menin-MLL complex components demonstrated the most extensive transcriptional effects, explaining their isolated and well-defined position in the UMAP. In contrast, the Polycomb complex perturbations had more limited effects, which was consistent with their distribution across a more diffuse, connected region (Fig. 1E, Supplementary Fig. 1D).

Having established a robust and biologically responsive system, we next explored the relationships between these transcriptional states. Pairwise correlation analysis highlighted strong coordination within the complexes (e.g., MEN1-DOT1L, r > 0.8). More importantly, this analysis revealed unexpected cross-complex synergy: the HAT component KAT6A exhibited significant positive correlations with MEN1 and DOT1L of the Menin-MLL complex (Supplementary Fig. 1E). Spatial expression patterns further localize Menin-MLL targets (e.g., *DOT1L, MEN1*) and *KAT6A* to discrete UMAP regions, with their density distributions aligning, further supporting cross-complex synergy (Fig. 1F).

### Computational modeling identified a clinically relevant myeloid differentiation program

**Fig. 2:**
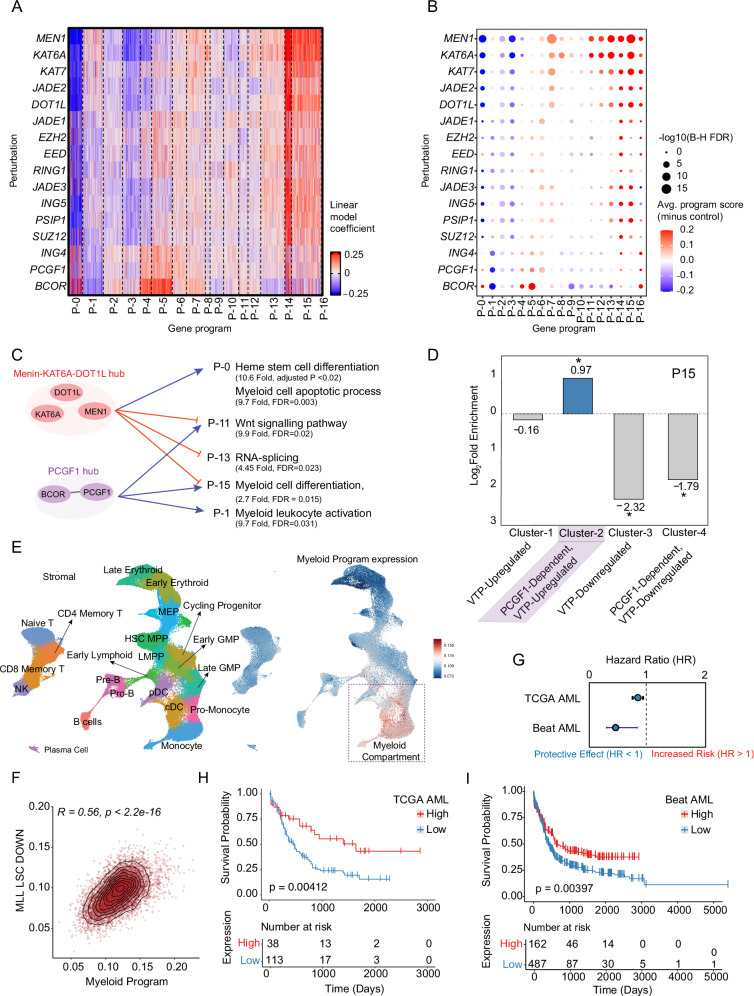
Computational modeling identified a clinically relevant myeloid differentiation program. **A** Heatmap showing the impact of epigenetic perturbations on the 17 gene programs. Red represents positive values (program upregulation following gene knockout), while blue represents negative values (program downregulation following gene knockout). **B** Bubble plot showing the enrichment of gene programs for each perturbation. The size of the bubbles represents the significance (-log_10_ FDR from two-sided *t*-test with Benjamini–Hochberg correction), and the color indicates the average program score relative to the control. **C** Functional enrichment analysis of the identified gene programs. **D** Barplot showing enrichment of differentiation-promoting gene signature in gene program P-15 (Myeloid Program). **E** UMAP projection of normal human bone marrow cells, colored by the Myeloid Program activity score, showing highest expression in mature myeloid lineages. **F** Scatter plot comparing the Myeloid Program score (x-axis) with MLL-LSC Down signature (y-axis). **G** Forest plots of hazard ratios (HR) for the Myeloid Program in the TCGA (top) and Beat-AML (bottom) cohorts. HRs were calculated using Cox proportional hazards regression models, adjusted for age, cytogenetic risk group, and mutation status. Error bars represent 95% confidence intervals. **H** Kaplan–Meier survival analysis of patients in the TCGA AML cohort (*n* = 151), stratified by high versus low Myeloid Program. **I** Kaplan–Meier survival analysis of patients in the Beat AML cohort (*n* = 649), stratified by high versus low Myeloid Program.

To characterize the transcriptional architecture and epigenetic perturbation effects, we used a regularized linear model to estimate perturbation impacts on highly variable genes, identifying 17 distinct transcriptional programs (designated P-0 to P-16, distinct from any individual genes with similar nomenclature) as coherent modules differentially regulated by specific perturbations. (Fig. 2A, B, Supplementary Table 3). Functional enrichment analyses revealed that the gene programs regulate crucial cellular processes associated with leukemogenesis. Program P-0 was significantly enriched for hematopoietic stem cell differentiation (fold enrichment 10.6, adjusted *P* < 0.05) and the myeloid cell apoptotic process (fold enrichment 9.7, adjusted *P* < 0.05); Program P-1 was significantly enriched for myeloid leukocyte activation (fold enrichment 4.14, adjusted *P* < 0.05); Program P-11 was significantly enriched for positive regulation of the Wnt signaling pathway (Fold enrichment 8.9, adjusted *P* < 0.05); Program P-13 was significantly enriched for RNA splicing pathways (fold enrichment 4.45, adjusted *P* < 0.05); P-15 was significantly enriched for myeloid cell differentiation (fold enrichment 2.7, adjusted P < 0.05), mitotic cell cycle phase transition (fold enrichment 4.2, adjusted *P* < 0.05), and the PI3K/AKT signaling pathway (specifically positive regulation of phosphatidylinositol 3-kinase/protein kinase B signal transduction; fold enrichment 3.47, adjusted *P* < 0.05) (Fig. 2C, Supplementary Table 4).

Among these modules, program P-15 stood out because of its strong enrichment of genes involved in myeloid cell differentiation, a process fundamentally disrupted in AML. Hereafter, we designate program P-15 as the ‘Myeloid Program’ for all subsequent analyses. *MEN1, DOT1L*, and *KAT6A* perturbations showed a coordinated effect on the Myeloid Program, with all three regulators negatively modulating this transcriptional module (Fig. 2B). *MEN1* perturbation impacted it by a magnitude of 0.43 (adjusted *P* = 1.18E−12), *DOT1L* perturbation by 0.27 (*p* = 0.00096), and *KAT6A* perturbation by 0.42 (*p* = 1.3E−07). This indicates that knockout of these genes results in increased Myeloid Program gene expression, suggesting they normally repress the Myeloid Program signature in AML cells under baseline conditions. Based on this shared regulatory function and transcriptional synergy, these factors are a “hub” that collaborates to control the Myeloid Program. We term this the “Menin-KAT6A-DOT1L hub” and hypothesize it represses the Myeloid Program through synergistic activity (Fig. 2C). In contrast, the perturbation of PRC1.1 components like *PCGF1* had a more subtle effect. While its direct impact on the Myeloid Program score appeared minimal in our initial model (Fig. 2B), it suggested a complex, non-synergistic role that we explore later.

To establish the biological significance of Myeloid Program, we investigated its overlap with known functional gene sets. Myeloid Program showed significant enrichment for a pro-differentiating gene signature that we previously reported (designated “Cluster 2”) [9], which is activated by Menin inhibition in a *PCGF1*-dependent manner (Fig. 2D). To further evaluate the relevance of the Myeloid Program to hematopoietic differentiation, we projected its activity score onto a UMAP of normal human bone marrow single-cell data from the Zeng et al. reference atlas [18]. This visualization revealed highest Myeloid Program activity in mature myeloid lineages, including monocytes and granulocyte-macrophage progenitors (GMPs), supporting its role in myeloid differentiation (Fig. 2E). We also assessed its relationship with a known leukemic stem cell (LSC) signature [19]. We found a strong positive correlation (R = 0.56, *p* < 2.2e−16) between the Myeloid Program score and a signature of genes downregulated in MLL-rearranged LSCs (Fig. 2F). This demonstrates that activation of the Myeloid Program is associated with a reduction in the leukemic stem cell state, reinforcing its pro-differentiation function opposing leukemogenesis.

Given that the Myeloid Program represents a pro-differentiation program whose repression is critical for leukemic maintenance, we also hypothesized that its baseline activity would correlate with patient outcomes. We tested this in two large, independent clinical cohorts. In the TCGA AML dataset (*n* = 151), elevated Myeloid Program activity predicted significantly improved overall survival (HR = 0.91, 95% CI: 0.86–0.96, adjusted *P* = 0.0017). This protective association was validated in the larger Beat AML dataset (*n* = 672), where higher Myeloid Program expression linked to better survival (HR = 0.29, 95% CI: 0.12–0.72, adjusted *P* = 0.046). Importantly, this association held true in both datasets after adjustment for known prognostic factors including age, cytogenetic risk, and mutation status (Fig. 2G). Of note, a comprehensive analysis of all 17 gene programs confirmed that P-15 was unique; most program did not show a statistically significant and consistent prognostic value across both the TCGA and Beat AML cohorts (Supplementary Fig. 2A, B). This protective association mirrored its pro-differentiation transcriptional state, further validating Myeloid Program as a conserved indicator of myeloid maturation and robust prognostic biomarker. To visualize this survival difference, patients were stratified into high and low Myeloid Program expression groups. Kaplan–Meier analysis confirmed these findings, showing a survival advantage for patients with high Myeloid Program expression in both the TCGA cohort (*p* = 0.00412; Fig. 2H) and the Beat AML cohort (*p* = 0.00397; Fig. 2I). To establish that the Myeloid Program (P-15) represents a distinct and clinically meaningful leukemic state rather than a nonspecific differentiation signal, we performed a permutation analysis using the MLL LSC-down gene set, which is the signature most closely correlated with P-15. We generated 10,000 random gene sets matching the size of the Myeloid Program and calculated their prognostic Hazard Ratios (HR) in the TCGA and Beat AML cohorts. The results showed that the observed prognostic HR of the Myeloid Program (HR = 0.91 in TCGA; HR = 0.29 in Beat AML) fell in the extreme low end of the distribution of HRs calculated from the random differentiation signatures (Supplementary Fig. 2C, D). This confirms that Myeloid Program (P-15) captures a unique, highly specific prognostic signal that differentiates it from generic differentiation signatures and confirms it as a robust indicator of favorable outcomes in AML.

Further stratification by molecular subtypes showed that Myeloid Program expression was higher in NPM1-mutated AML (TCGA: mean Δ = 1.14, *P* < 0.05, *n* = 36 with mutation vs. *n* = 115 wild-type); Beat AML: Δ = 1.91, *P* < 0.05, *n* = 152 with mutation vs. *n* = 520 wild-type), a genotype linked to chemotherapy sensitivity and favorable prognosis (Supplementary Fig. 2E). In contrast, TP53-mutated cases exhibited profound Myeloid Program suppression (Beat AML: mean Δ = −0.46, *P* < 0.05, *n* = 48; TCGA: Δ = −0.53, *P* < 0.05, *n* = 18), aligning with the therapy-resistant, undifferentiated phenotype characteristic of this high-risk subgroup (Supplementary Fig. 2F). Clinically, when stratified by disease stage in the Beat AML cohort, Myeloid Program was predominantly expressed in remission-stage patients but significantly downregulated in relapsed and residual disease (Supplementary Fig. 2G). Moreover, in the European Leukemia Net (ELN) classification analysis, Myeloid Program was among the most significantly enriched programs in favorable-risk AML patients, clearly distinguishing them from adverse-risk cases (Supplementary Fig. 2H). This consistent association between Myeloid Program, NPM1-mutated AML, and remission status across independent datasets strongly suggests that it functions as a myeloid differentiation pathway that contributes to favorable clinical outcomes when derepressed. Collectively, these results define gene Program P-15 as a clinically significant myeloid differentiation program, revealing its repression by a novel epigenetic hub, explaining a key mechanism of therapeutic resistance, and validating its activity as a powerful predictor of patient survival.

### Targeting the MEN1/DOT1L-KAT6A hub reveals a potent synergistic vulnerability in AML

**Fig. 3:**
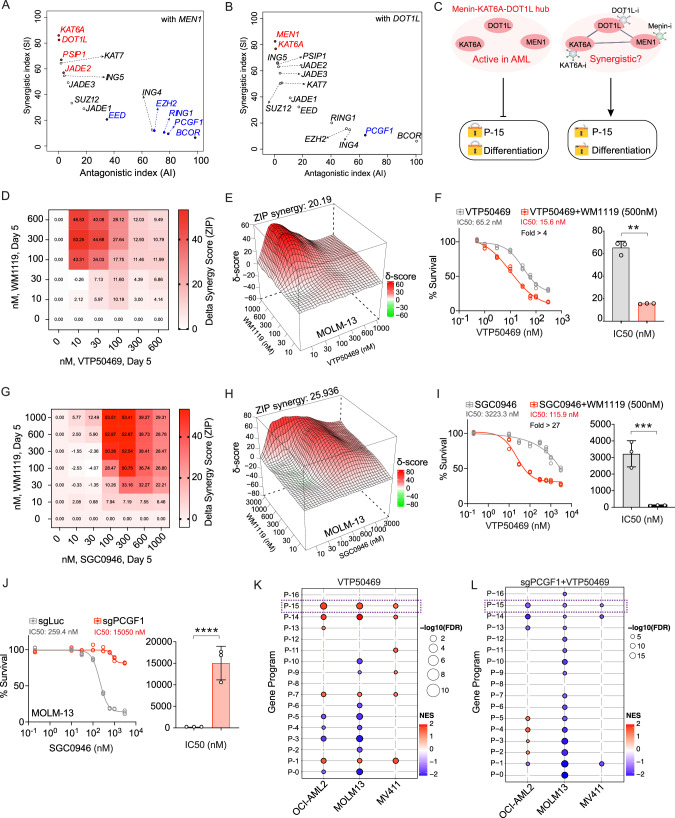
Targeting the MEN1/DOT1L-KAT6A hub reveals a potent synergistic vulnerability in AML. Antagonistic and Synergistic Index (AI, SI) scores for MEN1 (**A**) and DOT1L (**B**) perturbation against other targets. The x-axis represents the Antagonistic Index (AI), and the y-axis represents the Synergistic Index (SI). Scores were calculated based on weighted similarity matrices incorporating protein-protein interaction networks from STRING database, with higher values indicating stronger predicted interactions. **C** Schematic model illustrating the proposed mechanism where the Menin-KAT6A-DOT1L hub cooperatively represses the Myeloid Program (P-15) to block differentiation. Pharmacological inhibitors targeting the hub are shown. **D** Delta synergy score heatmap showing the combination effects of VTP50469 (Menin inhibitor) and WM1119 (KAT6A inhibitor) in MOLM-13 cells. Color intensity represents magnitude of synergy (red) or antagonism (blue) at each concentration combination. **E** ZIP synergy score heatmap for the VTP50469 and WM1119 combination, with an overall synergy score of 20.19. **F** Dose–response curve (left) and corresponding IC_50_ comparison (right) for VTP50469 alone versus in combination with WM1119 (500 nM). Data represent the mean ± standard deviation from three biological replicates (****p < 0.0001). **G** Delta synergy score heatmap for the combination of the DOT1L inhibitor (SGC0946) and the KAT6A inhibitor (WM1119) in MOLM-13 cells. **H** ZIP synergy score heatmap for the SGC0946 and WM1119 combination, with an overall synergy score of 25.94. **I** Dose–response curve (left) and corresponding IC_50_ comparison (right) for SGC0946 alone versus in combination with WM1119. Data represent the mean ± standard deviation from three biological replicates. **J** Dose–response curve (left) and IC_50_ comparison (right) demonstrating that knockout of PCGF1 (sgPCGF1) confers resistance to the DOT1L inhibitor SGC0946 compared to control cells (sgLuc). **K** Gene Set Enrichment Analysis (GSEA) showing uniform activation of the Myeloid Program (P-15) across three AML cell lines (OCI-AML2, MOLM13, MV4-11) following treatment with a Menin inhibitor. **L** GSEA showing repression of the Myeloid Program across the same three cell lines when treated with a Menin inhibitor in a PCGF1 knockout background, demonstrating PCGF1’s role in mediating the drug response.

Based on our model proposing that Myeloid Program is co-regulated by distinct epigenetic hubs, we next sought to functionally validate the predicted interactions and explore their therapeutic potential. To achieve this, we first employed a computational approach to quantitatively assess potential synergistic and antagonistic interactions, we calculated weighted interaction indices based on gene signatures related to cell cycle regulation, DNA damage response, and apoptosis (Supplementary Fig. 3A). This analysis identified strong synergy between *MEN1* and *KAT6A* perturbations (Synergistic Index = 86, in a range of none at 0 to full at 100), affecting the Myeloid Program (Fig. 3A). In contrast, *DOT1L* and *PCGF1* demonstrated strong antagonistic interaction (Antagonistic Index = 63), with *PCGF1* perturbation counteracting the transcriptional effects of *DOT1L* disruption (Fig. 3B). This antagonistic relationship suggests a regulatory balance between activating and repressive epigenetic complexes working in concert to maintain precise control over differentiation programs.

Based on our Perturb-seq and clinical correlation analyses, we hypothesized that simultaneously targeting the KAT6A-Menin-DOT1L hub could effectively disrupt leukemic transcriptional programs, particularly affecting the clinically relevant Myeloid Program (Fig. 3C). Pharmacological targeting of the KAT6A-Menin-DOT1L hub could disrupt the epigenetic repression of Myeloid Program, offering a rational approach to restore differentiation in AML. To test this hypothesis, we conducted combination inhibition experiments using selective small molecule inhibitors: VTP50469 (Menin-MLL inhibitor) [20], WM1119 (KAT6A inhibitor) [21], and SGC0946 (DOT1L inhibitor) [22]. In KMT2A-rearranged MOLM-13 (KMT2A-AF9) cells, the combination of VTP50469 and WM1119 yielded a ZIP synergy score of 20.19, reducing the IC_50_ of the Menin inhibitor from approximately 65 nM to 15 nM (Fig. 3D-F). Importantly, this synergistic vulnerability was conserved across genetically distinct KMT2A-rearranged subtypes. In the MV4-11 model (KMT2A-AF4), the combination treatment showed a strong synergistic effect (ZIP synergy score: 15.635) and reduced the IC_50_ from ~520 nM to 150 nM (Supplementary Fig. 3B, C). Similarly, OCI-AML2 cells (KMT2A-AF6) displayed robust synergy (ZIP synergy score: 10.203), with the IC_50_ reducing from ~2200 nM to 450 nM (Supplementary Fig. 3D, E). Together, these results indicate that dual targeting of Menin and KAT6A consistently potentiates differentiation-associated responses across KMT2A-rearranged AML models, consistent with coordinated disruption of the epigenetic repression that maintains the Myeloid Program.

Similarly, co-inhibition of DOT1L (SGC0946) and KAT6A (WM1119) demonstrated synergistic effects on cell viability (Fig. 3G). The combination produced a ZIP synergy score of 25.94, supporting the effectiveness of targeting multiple components of the KAT6A-Menin-DOT1L hub (Fig. 3H). Dose–response curves showed that the combination treatment decreased cell viability compared to SGC0946 (DOT1L inhibitor) alone, reducing the IC_50_ from around 3000 nM to 100 nM (*p* < 0.05) (Fig. 3I). Our model’s prediction of an antagonistic interaction between DOT1L and PCGF1 was also functionally validated at the level of therapeutic response. We observed that PCGF1 disruption (sgPCGF1) conferred resistance to the DOT1L inhibitor SGC0946, leading to an increased IC_50_ from around 250 nM to 15000 nM (*p* < 0.05) (Fig. 3J). Furthermore, PCGF1 knockout conferred significant resistance to SGC0946 in both MV4-11 (Supplementary Fig. 3F) and OCI-AML2 (Supplementary Fig. 3G), supporting our model that antagonistic epigenetic interactions between PRC1.1 components and DOT1L contribute to resistance to DOT1L inhibition. This finding aligns with our Perturb-seq data showing antagonistic effects between DOT1L and PCGF1 perturbations, particularly in regulation of cell cycle genes, and suggests a potential mechanism of resistance to DOT1L inhibitors through compensatory activity of the PRC1 complex. This antagonism further supports the model of antagonistic epigenetic crosstalk, where disruption of one component of the regulatory balance can be offset by modulation of an opposing factor.

Finally, to confirm that these therapeutic outcomes are directly mediated by the Myeloid Program and are generalizable across different AML contexts, we performed Gene Set Enrichment Analysis (GSEA) on transcriptional data from three distinct AML cell lines. The analysis confirmed that Myeloid Program was uniformly and significantly activated by VTP treatment across OCI-AML2 (NES = 2.07), MOLM13 (NES = 2.01), and MV4-11 (NES = 1.65) cell lines (all FDR < 2.2 × 10⁻⁴), reflecting a robust and reproducible activation signature driven by Menin-MLL inhibition (Fig. 3K). In direct opposition, Menin inhibition but *PCGF1* knockout resulted in the consistent and significant repression of Myeloid Program across all three lines: OCI-AML2 (NES = –1.92), MOLM13 (NES = –1.51), and MV4-11 (NES = –1.43) (all FDR < 2.5 × 10⁻⁴), reflecting the role of PRC1-mediated repression in maintaining P-15 suppression (Fig. 3L). This convergence across distinct cell lines and independent epigenetic perturbations reinforces the mechanistic relevance of the Myeloid Program and underscores its generalizability as a robust drug-responsive transcriptional module.

### Dual inhibition of KAT6A and MEN1 synergistically remodels the transcriptome to activate a pro-differentiation state

**Fig. 4:**
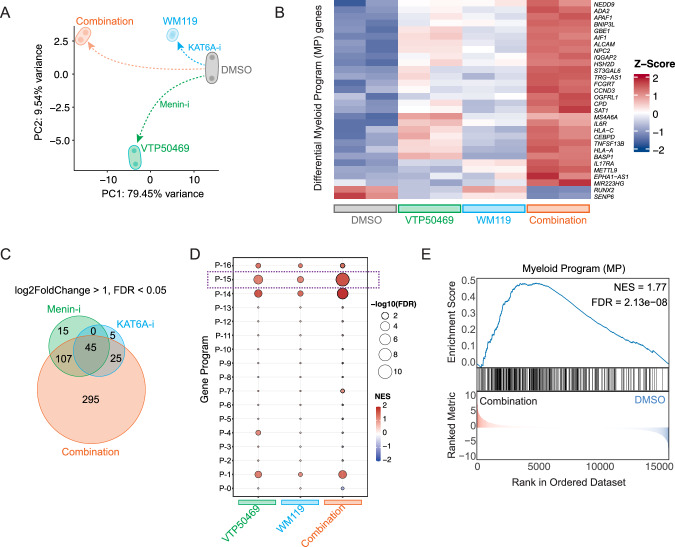
Dual inhibition of KAT6A and MEN1 synergistically remodels the transcriptome to activate a pro-differentiation state. **A** Principal Component Analysis (PCA) of bulk RNA-seq data from MOLM-13 cells treated with DMSO, WM1119 (KAT6A-i), VTP50469 (Menin-i), or the combination. **B** Heatmap showing Z-scores of differentially expressed genes across treatment conditions. **C** Venn diagram illustrating the overlap of significantly upregulated genes across treatment conditions. Significance was defined as Log_2_Fold Change > 1 and FDR < 0.05. **D** Dot plot showing Gene Set Enrichment Analysis (GSEA) for all 17 transcriptional programs (y-axis) across single and combination treatments (x-axis). Dot size represents statistical significance (−log_10_FDR), and dot color indicates the Normalized Enrichment Score (NES). **E** GSEA enrichment plot comparing the combination treatment (WM1119 + VTP50469) against the DMSO control.

Having established the clinical relevance of the Myeloid Program and validated the predicted synergy between *MEN1* and *KAT6A* using cell viability assays, we next sought to uncover the transcriptional mechanism driving this synergy. To achieve this, we performed bulk RNA sequencing on MOLM-13 cells following treatment with MEN1 inhibitor (VTP50469), KAT6A inhibitor (WM1119), or the combination. Principal Component Analysis (PCA) of the transcriptomes showed a profound and distinct impact of the dual inhibition. The treatment groups separated clearly, with the combination treatment forming a unique cluster far from both the single agents and the DMSO control (Fig. 4A). The first two principal components accounted for nearly 90% of the total variance (PC1: 79.45%, PC2: 9.54%), confirming that the combination therapy induces a unique transcriptional state not achievable with either drug alone.

To understand the key drivers of this unique state, we first visualized the expression of the differentially expressed genes belonging to the Myeloid Program signature. A heatmap clearly illustrated the synergistic effect at the gene level, showing that while single agents caused modest changes, the combination treatment led to strong and uniform upregulation of core Myeloid Program member genes, including myeloid regulators like *CEBPD* and *ALCAM* (Fig. 4B). We then quantified the extent of this synergy by analyzing the overlap of all significantly upregulated genes across conditions. A Venn diagram revealed that the combination therapy activated a large, distinct set of genes, with 295 genes being upregulated exclusively by the dual treatment (Fig. 4C).

Finally, pathway-level analysis confirmed that the Myeloid Program was the most significantly enriched pathway in the dual-inhibitor-treated cells (Fig. 4D, E). The primary Gene Set Enrichment Analysis (GSEA) plot demonstrated a robust and highly significant positive enrichment for the Myeloid Program gene set when comparing the combination treatment to the control (NES = 1.77, *p*-adj = 2.13e−08) (Fig. 4E). While the single-agent treatments also induced Myeloid Program, their effect was substantially less pronounced (NES = 1.35 for Menin-i; NES = 1.28 for KAT6A-i) (Supplementary Fig. 4A, B). Together, these results provide transcriptional evidence that dual targeting of MEN1 and KAT6A synergistically disrupts epigenetic repression to promote myeloid differentiation.

### The Myeloid Program score predicts drug sensitivity and identifies novel synergies with Menin inhibition

With the Myeloid Program established as a robust prognostic biomarker and a key mechanistic node in AML, we next sought to determine if its baseline activity could predict sensitivity to other therapeutic agents. We hypothesized that the transcriptional state defined by high Myeloid Program activity, which is characteristic of more differentiated phenotype, might create unique pharmacological vulnerabilities.

**Fig. 5:**
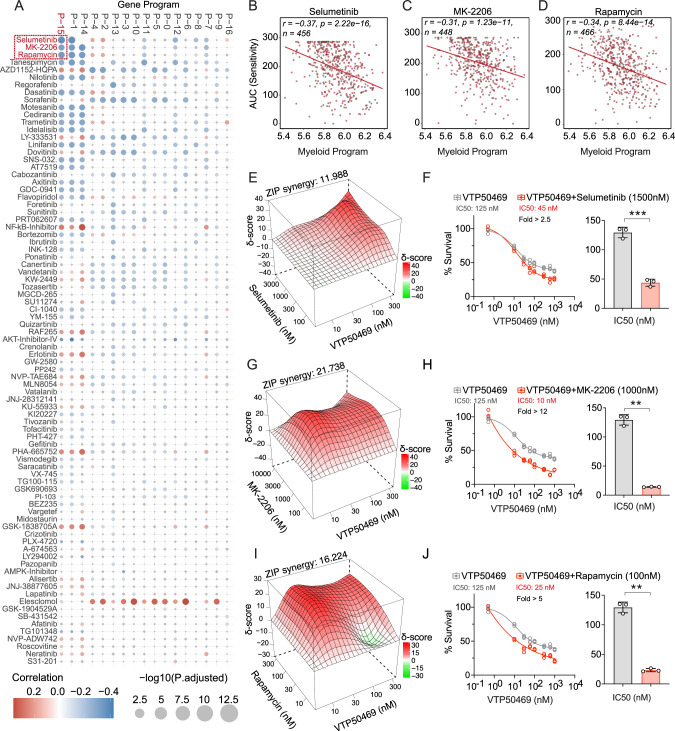
The Myeloid Program score predicts drug sensitivity and identifies novel synergies with Menin inhibition. **A** Correlation plot of gene program activity and drug sensitivity (measured by area under the curve, AUC) in the Beat AML cohort. Color indicates the Pearson correlation coefficient (blue denotes negative correlation, corresponding to lower AUC and therefore higher drug sensitivity in leukemias with higher gene program activity), and point size reflects statistical significance (−log_10_ adjusted *P* value). Scatter plots showing the significant correlation between Myeloid Program activity and sensitivity to Selumetinib (MEK inhibitor) (**B**), Rapamycin (mTOR inhibitor) (**C**) and MK-2206 (AKT inhibitor) (**D**). **E** ZIP synergy scores for the combination of VTP50469 with Selumetinib. **F** Dose–response curves showing significant reduction in the IC_50_ of VTP50469 when combined with Selumetinib. **G** ZIP synergy scores for the combination of VTP50469 with MK-2206. **H** Dose–response curves showing significant reduction in the IC_50_ of VTP50469 when combined with MK-2206. **I** ZIP synergy scores for the combination of VTP50469 with Rapamycin. **J** Dose–response curves showing significant reduction in the IC_50_ of VTP50469 when combined with Rapamycin.

To investigate this, we correlated the Myeloid Program score with drug sensitivity data quantified by the Area Under the Curve (AUC) from the Beat AML cohort (Fig. 5A). This analysis showed a negative correlation between Myeloid Program activity and AUC of inhibitors targeting specific signaling pathways. Because a lower AUC corresponds to higher drug sensitivity, these results suggested that elevated Myeloid Program activity predicts sensitivity to the MEK inhibitor Selumetinib (Pearson’s r = −0.37, *p* = 2.2e−16), the mTOR inhibitor Rapamycin (r = −0.34, *p* = 8.44e−14), and the AKT inhibitor MK-2206 (r = −0.31, *p* = 1.2e−11) (Fig. 5B, D). This trend was also observed for other compounds, including the VEGFR inhibitor Cediranib (Supplementary Fig. 4C).

These data reveal a state-dependent vulnerability in which induction of the Myeloid Program may define a cellular state that is selectively sensitive to specific signaling pathway inhibition. We therefore hypothesized that co-treatment with the top-ranked drugs from our screen and the Menin inhibitor VTP50469, which induces the Myeloid Program, could result in synergistic therapeutic effects. Combination treatment of VTP50469 with Selumetinib in MOLM-13 resulted in a synergistic effect, with a Zero Interaction Potency (ZIP) synergy score of 11.988 (Fig. 5E). This combination significantly reduced the IC_50_ of VTP50469 from ~125 nM to 45 nM (*P* < 0.001) (Fig. 5F). We next confirmed strong Menin-KAT6A synergy in the KMT2A-rearranged MV4-11 model, where co-treatment produced a ZIP synergy score of 15.527 and reduced the IC_50_ of VTP50469 to below 40 nM (Supplementary Fig. 4D, E). We observed similar enhancement with the AKT inhibitor MK-2206 which showed a ZIP synergy score of 21.738 (Fig. 5G) and reduced the IC_50_ of VTP50469 to below 10 nM in MOLM-13 (Fig. 5H), while in MV4-11 showed the ZIP synergy score of 11.69 and reduced IC50 of VTP50469 to below 160 nM (Supplementary Fig. 5F, G). The combination with Rapamycin was also synergistic, with a ZIP score of 16.224 (Fig. 5I), and reduced the IC_50_ of VTP50469 to approximately 25 nM in MOLM-13 (Fig. 5J). Other compounds like Cediranib also had similar synergy with VTP50469 (Supplementary Fig. 4H, I). Taken together, these results support the Myeloid Program as a predictive biomarker for drug sensitivity and provide a rationale for combining Menin inhibition with pathway-targeted agents to enhance anti-leukemic efficacy.

Finally, the Myeloid Program also predicted therapeutic interactions involving regulators beyond the initial perturbation screen. To distinguish circuit-specific cooperation from broader differentiation effects, we evaluated BRD4 (not included in the original Perturb-seq screen) and EZH2 (included in the screen) as both are known to confer differentiation upon perturbation [23–26]. Publicly available data (GSE29799) showed that BRD4 inhibition strongly activates the Myeloid Program (GSEA NES = 1.91, FDR = 3.25e−11) (Supplementary Fig. 4A). Consistent with this convergence, combining the Menin inhibitor VTP50469 with the BRD4 inhibitor JQ1 produced synergy in MOLM-13 and an additive interaction in MV4-11 (Supplementary Fig. 5B, C). In contrast, EZH2 perturbation did not significantly activate the Myeloid Program in our Perturb-seq dataset, and our model accordingly predicted an antagonistic interaction with Menin inhibition. Experimentally, VTP50469 combined with the EZH2 inhibitor GSK126 resulted in antagonism (ZIP scores: −4.1 in MOLM-13 and −6.1 in MV4-11) (Supplementary Fig. 5D, E). Together, these divergent patterns demonstrate that P-15 distinguishes hub-specific cooperation from broader, nonspecific differentiation effects.

## Discussion

We used integrated computational modeling of high-throughput Perturb-seq to deconstruct the epigenetic dependencies of KMT2A-rearranged AML, leading to the identification of a critical transcriptional module we term the Myeloid Program. This transcriptional module, which we identified through co-regulation, is notably enriched with genes pivotal for myeloid maturation and function, including key surface markers (e.g., *CD14*, *CD33*, *ITGAM*), enzymes crucial for myeloid effector functions (e.g., *MPO*, *LYZ*), and, significantly, master transcriptional regulators of myelopoiesis such as *CEBPA* and *SPI1* (PU.1). While Menin-MLL inhibition has previously been shown to induce differentiation in MLL-rearranged leukemia models [20], and DOT1L’s role in maintaining leukemic stem cells through H3K79 methylation has been well-documented [27], we found KAT6A to be a part of a regulatory network converging to repress a shared differentiation program, aligning with emerging models of “epigenetic cooperativity” in AML [28], where multiple chromatin modifiers act in concert to enforce stemness.

Our analysis demonstrates that MEN1, DOT1L, and KAT6A repress Myeloid Program, with their perturbations leading to significant derepression of this program. The synergy between MEN1 and KAT6A, evidenced by their pharmacological inhibition, suggests a cooperative mechanism that amplifies Myeloid Program repression. As a validation, several predictions of interactions from the method have been documented in previously published results [9, 29]. For example, KAT6A inhibitor (WM1119) has been shown to enhance sensitivity to SNDX-50469, a Menin inhibitor [29]. Similarly, *PCGF1* knockout, *BCOR* knockout, *EZH2* knockout and *EED* knockout individually leads to resistance to Menin inhibitor response [9]. KAT6A has been implicated in maintaining MLL-rearranged AML through acetylation of H3K23, and our finding that dual inhibition of KAT6A and Menin synergistically activates differentiation pathways mirrors observations of enhanced efficacy when combining epigenetic therapies (e.g., EZH2 and BET inhibitors; [30, 31]. Recently, it has been shown that KAT6A inhibition may exhibit limited therapeutic efficacy in KMT2A-r AML [10]. Consistent with this observation, our dose–response curves in OCI-AML2 indicate that WM1119 (KAT6A-i) alone has minimal cytotoxicity compared to VTP50469 (Menin-i) (Supplementary Fig. 5F). Importantly, this limited single-agent activity is fully aligned with our core finding that the synergistic co-inhibition of KAT6A and MEN1 can overcome the functional redundancy and compensatory mechanisms of the Menin-KAT6A-DOT1L hub. Concurrent studies have independently validated this approach, demonstrating that catalytic inhibition of KAT6/KAT7 overcomes both primary and acquired resistance to Menin inhibitors in MLL leukemia [32]. This suggests that targeting nodes within interconnected epigenetic networks may overcome the functional redundancy that often limits single-agent efficacy. Overall, we observe that KAT6A, Menin, and DOT1L cooperatively repress the differentiation-associated Myeloid Program in AML cells, thereby maintaining leukemic state by blocking cellular differentiation. Intriguingly, the therapeutic potential of this epigenetic co-dependency may extend beyond hematological malignancies, as combined inhibition of KAT6A/B and Menin has also been shown to reverse oncogenic gene expression programs in models of breast cancer [33]. Interestingly, the Menin–KAT6A inhibitor combination also displayed robust synergy in the non-KMT2A-rearranged, NPM1-mutated cell line OCI-AML3 (ZIP synergy score 12.8) (Supplementary Fig. 4G). This observation is consistent with emerging clinical data showing activity of Menin inhibitors in NPM1-mutated AML and suggests that the Menin–KAT6A–DOT1L repressive hub and its control of the Myeloid Program extend beyond KMT2A rearrangements to other differentiation-blocked genotypes that rely on similar epigenetic circuitry. These results broaden the potential therapeutic relevance of co-targeting Menin and KAT6A beyond KMT2A-rearranged AML.

In contrast to this synergy, our model showed a critical antagonistic relationship between the activating complex component *DOT1L* and the Polycomb Repressive Complex 1.1 component PCGF1. We functionally confirmed this prediction, showing that disruption of *PCGF1* confers significant resistance to *DOT1L* inhibition. This finding provides a compelling mechanistic explanation for therapeutic resistance, wherein the loss of a PRC1.1 component can compensate for the pro-differentiating effects of targeting the Menin-MLL/DOT1L hub. This aligns with recent reports that *PCGF1* loss confers resistance to Menin inhibitors [9], and our work expands this principle to *DOT1L* inhibitors. This regulatory balance, where the Myeloid Program is co-repressed by antagonistic epigenetic forces, shows the complexity of leukemia maintenance and suggests that durable therapeutic responses will require strategies that disrupt multiple arms of this balanced network.

Although the individual roles of Menin, DOT1L, and KAT6A in sustaining HOX/MEIS1-driven programs and blocking differentiation in KMT2A-rearranged and NPM1-mutated AML have been extensively documented [3, 5, 27, 34, 35], the higher-order regulatory relationships among these factors have been less clear. Prior studies necessarily focused on one node at a time and could not resolve whether limited clinical responses to single-agent Menin, DOT1L, or KAT6A inhibitors reflected incomplete target engagement, off-target effects, or compensatory mechanisms within a broader chromatin network. By applying Perturb-seq to systematically map functional interactions among core chromatin regulators, we show that Menin, KAT6A, and DOT1L operate as a cooperative repressive hub that jointly silences a shared myeloid differentiation program, while PRC1.1 (PCGF1) acts as a critical antagonistic node. This hub designation reflects functional co-regulation supported by correlated transcriptional consequences and synergistic drug response and provides a mechanistic framework for understanding both the partial efficacy of monotherapy and the potent, reproducible synergy of dual Menin-KAT6A inhibition across genetically diverse AML models.

We also found a clinical relevance of the Myeloid Program by its robust prognostic value across independent AML cohorts (TCGA and Beat AML). We demonstrate that high baseline activity of this program is a powerful and independent prognostic factor for improved overall survival. Its expression robustly stratifies patients, aligning with known clinical subtypes; the program is significantly elevated in favorable-risk, NPM1-mutated AML and suppressed in aggressive, TP53-mutated cases. Furthermore, its activity is higher in patients in remission compared to relapsed, confirming its association with a less malignant, differentiated state. These findings position Myeloid Program as a conserved biomarker of myeloid maturation, with potential utility in clinical stratification and risk assessment.

Beyond its prognostic value, we establish the Myeloid Program as a predictive biomarker that can guide therapeutic strategy. By correlating the program’s activity with drug sensitivity data from the Beat AML cohort, we show a vulnerability that AML cells with a high Myeloid Program score (representing differentiated state) are uniquely sensitive to inhibitors of the PI3K/AKT/mTOR pathway and the MEK inhibitor Selumetinib [36]. This finding is supported by our observation that the Myeloid Program is itself enriched for genes that control these signaling axes like PI3K/AKT (e.g., positive regulation of phosphatidylinositol 3-kinase/protein kinase B signal transduction) and MAPK signaling components. This suggests that as leukemia cells transition away from an immature state, they begin to rely on these specific signals to survive (Supplementary Table 4) [37, 38]. Consequently, this relationship supports a differentiation-primed sensitization strategy for combination treatment. In this approach, agents that induce differentiation (such as Menin inhibitors, which activate the Myeloid Program) are used to drive immature cells into a state that is then selectively vulnerable to the inhibition of these essential signaling pathways. This data-driven framework provides a rational basis for designing combination therapies that target distinct cellular states within a heterogeneous tumor.

Although our findings are validated across multiple in vitro KMT2A models, in vivo evaluation and primary patient studies will be essential. Given the potential for differentiation-based therapies to induce systemic toxicities, comprehensive efficacy and safety assessment in PDX models, especially derived from poor prognosis, low-Myeloid Program AML, should be prioritized. Mechanistic studies defining how Menin, KAT6A, and DOT1L assemble into a repressive module, including chromatin profiling showing co-occupancy and biochemical evidence of physical interaction, and how additional regulators such as BRD4 interface with this circuitry, will further refine our model. High-dimensional combinatorial perturbation strategies, including pooled dual-sgRNA CRISPR screens [39], will be powerful for mapping the broader interaction landscape that shapes this epigenetic circuit.

In conclusion, our study identifies a critical epigenetic hub that maintains leukemic stemness by repressing a clinically relevant Myeloid Program. We demonstrate that targeting this hub with combination therapies offers a potent, synergistic strategy to overcome epigenetic redundancy and induce differentiation. Finally, we identify the Myeloid Program as a biomarker that is both prognostic for patient survival and predictive of novel therapeutic vulnerabilities. This work provides a rational framework for designing new combination therapies and stratifying patients, for more durable and personalized treatments for AML.

## Materials and methods

### Cell lines

Human embryonic kidney 293FT cells (HEK293FT), used for lentiviral production, were cultured in Dulbecco’s Modified Eagle Medium (DMEM, Gibco) supplemented with 10% fetal bovine serum (FBS, Gibco) and 1% Penicillin-Streptomycin (Gibco). The acute myeloid leukemia (AML) cell line MOLM-13, MV4-11, OCI-AML2 and OCI-AML3 were maintained in Roswell Park Memorial Institute (RPMI) 1640 medium (Gibco) enriched with 10% FBS and 1% Penicillin-Streptomycin. All cell lines were incubated at 37 °C in a humidified atmosphere containing 5% CO_2_. MSCV-Cas9-Puro plasmid (Addgene #65655) was employed for stable Cas9 expression in MOLM-13 cells as previously described [40]. All cell lines were tested free of mycoplasma contamination using MycoAlert PLUS Mycoplasma Detection Kit (Lonza).

### Small molecule inhibitors

The following small molecule inhibitors were used: VTP50469 (MedChemExpress, HY-114162), WM1119 (MedChemExpress, HY-102058), SGC0946 (MedChemExpress, HY-15650), Selumetinib (MedChemExpress, HY- 50706), Rapamycin (MedChemExpress, HY- 10219), MK-2206 dihydrochloride (MedChemExpress, HY-10358), Cediranib (MedChemExpress, HY-10205) and GSK126 (MedChemExpress, HY-13470). (S)-JQ1 was prepared as previously described [41].

### Cell viability assay

Cell viability assays were performed as previously described [42]. Cells were seeded in triplicate at a density of 20,000 cells/mL in 24-well plates and subjected to treatment of compounds at various concentrations. Medium containing fresh compound was changed every 3 days. Cell viability was determined with ATP-based luminescent viability assay using the CellTiter-Glo 2.0 reagent (Promega, G9242) according to the manufacturer’s instructions. The cell viability luminescent signal was measured using Synergy H1 Hybrid Reader (BioTek). IC_50_ values were calculated as previously described [43] using a nonlinear regression fit of log-scaled inhibitor concentration versus normalized response in GraphPad Prism v9. Drug synergy was quantified using the Zero Interaction Potency (ZIP) model. All synergy scores and 3D synergy plots were generated using the SynergyFinder (web application) to identify potent synergistic areas across the dose–response matrix.

### Plasmid construction and cloning

The Perturb-seq library was constructed using the pBA904 vector (Addgene plasmid #122238). The vector was first linearized using BstXI and BlpI restriction enzymes (New England Biolabs) according to the manufacturer’s instructions, creating a linear backbone suitable for cloning. Guide RNAs (sgRNAs) were designed to target 16 key chromatin regulators, including genes from the Menin-MLL, Polycomb, and HAT complexes. sgRNAs were designed using the Broad Institute’s sgRNA Designer. Oligonucleotides encoding the sgRNA sequences were synthesized, annealed, and ligated into the linearized pBA904 vector. Ligation reactions were transformed into NEB Stable Competent E. coli (New England Biolabs), and transformants were plated on LB-agar plates containing ampicillin (100 μg/mL). After overnight incubation at 37 °C, colonies were screened by colony PCR to verify the presence of the sgRNA inserts.

### Lentivirus generation and infection

Lentiviral particles were produced in HEK293FT cells co-transfected with the pBA904 sgRNA library plasmid, psPAX2 packaging plasmid, and pMD2.G envelope plasmid using the Nanofect transfection reagent. Viral supernatants were collected at 48- and 72-h post-transfection, pooled, and stored at −80 °C. For transduction, MOLM-13 cells were incubated with lentivirus in the presence of 5 µg/mL polybrene (Sigma) using a centrifugal infection protocol (spinfection) at 2400 RPM for 1.5 h at 25 °C.

### Fluorescence-activated cell sorting

Post-transduction, MOLM13 cell lines were prepared for fluorescence-activated cell sorting (FACS). Cells were resuspended in PBS supplemented with 2% fetal bovine serum to maintain viability and sorted using the BD FACSAria II system. The sorting criteria focused on isolating live cells with successful blue fluorescent protein (BFP) expression, indicating effective transduction.

### Single-cell RNA-seq

Approximately 43,750 BFP-positive MOLM-13 cells were loaded into a Chromium Controller (10× Genomics) for single-cell RNA sequencing. The generated libraries captured a total of 1.3 billion reads for gene expression analysis and 315 million reads for the feature barcode library, providing comprehensive coverage and depth for subsequent bioinformatic analysis.

### Perturb-seq data pre-processing

Single-cell RNA sequencing data were processed using Cell Ranger (v7.1.0) to generate a filtered gene expression matrix, aligned against the pre-built human reference genome GRCh38-2024-A. Quality control (QC), normalization, and scaling were performed using Seurat as previously described [44]. Captured guide RNAs were used to identify doublets and assign cells to their respective perturbations. Cells were filtered based on the following criteria: [1] cells with fewer than 200 detected genes were removed [2]. Cells with a total count below 1000 or more than 20% mitochondrial reads were excluded.

### Clustering and visualization

Dimensionality reduction, clustering and visualization were performed using Seurat v 5.4.0. To assess cell density on the UMAP, a density plot was generated using ggplot2.

### Differential gene expression analysis (Perturb-seq)

Differential expression analysis was performed for each gene by comparing perturbed cells to control cells (cells containing any of the three control guides). Only genes expressed in more than 50% of control cells were included in the analysis. Seurat’s Wilcoxon rank-sum test (non-parametric) was used to compute differential expression, with log2 fold change and false discovery rate (FDR)-adjusted *p*-values reported.

### RNA extracting, RNA-seq and data analysis

RNA was extracted from cells using the RNeasy kit (Qiagen) following the manufacturer’s instructions. RNA-seq libraries were generated using the NEBNext Ultra RNA Library Prep Kit for Illumina (New England Biolabs), following the manufacturer’s protocol. The quality and concentration of the libraries were examined by Bioanalyzer High Sensitivity DNA Chip (Agilent). The multiplexed RNA-Seq libraries were paired-end sequenced for 150 bp on the Illumina HiSeq 4000 platform. The paired-end reads were mapped to the human genome (hg38) using STAR (v2.5.1b) with default parameters. The read counts were used for DESeq2 (v4.5.2) to define differentially expressed genes as previously described [45]. Principal component analyses (PCA) were carried out for visualizing the reproducibility of sample replicates and the differences among distinct sample groups in the transcriptome. Log2-transformed expression values of all mRNAs were applied as features of PCA. The pairwise distances between features were derived through the Euclidean method. PCA was done by the R package prcomp [46]. Differential expression analyses comparing 4 sample groups, such as MOLM13 cells treated with DMSO, Menin inhibitor VTP50469, the KAT6A inhibitor WM1119, or a combination of VTP50469 and WM1119, were carried out using DESeq2 R package [47]. A cutoff of Benjamini–Hochberg adjusted *p* value 0.05 was applied to define DE mRNAs. Gene set enrichment analysis (GSEA) was performed using fgsea v 1.36.2 as previously described [42].

### Linear model for regulatory matrix inference

A linear model with elastic net regularization [48] was trained to infer the regulatory matrix. In summary, the model estimated the effect of each perturbation by minimizing the following objective function, incorporating both L1 (lasso) and L2 (ridge) regularization:

$$\min \left({{||}{{\mbox{y}}}-{{I}}_{\Delta }\cdot {{\rm{\beta }}}{||}}_{2}^{2}+\lambda \left(\alpha {{||}{{\rm{\beta }}}{||}}_{1}+\left(1-\alpha \right){{{\rm{||}}}{{\rm{\beta }}}{{\rm{||}}}}_{2}^{2}\right)\right)$$

where ${{\bf{y}}}$ is a vector of expression values, ${{\boldsymbol{\beta }}}$ is the regulatory coefficient vector, ${{{\boldsymbol{I}}}}_{{{\boldsymbol{\Delta }}}}$ is a binary indicator that is 1 for a perturbed cell, and 0 indicates an unperturbed cell, ${{\boldsymbol{\lambda }}}$ is a multiplier for regularization terms, and ${{\boldsymbol{\alpha }}}$ controls the balance between L1 (sparsity) and L2 (shrinkage).

For model training, the expression data was split into 80% training set, 10% test set, and 10% validation set. The regulatory coefficient matrix was constructed for perturbations across highly variable genes, defined by a minimum mean expression of 0.0125, a maximum mean of 3, and a minimum dispersion of 0.5.

### Gene programs

Following Otto et al. [49], gene programs were defined by clustering genes in the regulatory matrix obtained via linear regression as described above. Louvain clustering was applied to the regulatory coefficients, using a nearest-neighbor graph (*k* = 5) to group genes based on shared perturbation effects.

### Perturbation combination effect analysis

Perturbation combination effect analysis followed the approach of Cheng et al. [50] by quantifying the overlap of targets within cell fitness-related pathways, including apoptosis, cell cycle regulation, and checkpoint control. To account for potential interrelatedness among genes, we incorporated a protein-protein interaction (PPI) network extracted from STRINGdb, represented by a similarity matrix Ω.

The similarity between two perturbations, ${{\boldsymbol{S}}}\left({{\bf{a}}},{{\bf{b}}}\right)$, was measured using an inner product defined on the gene pathway space:

$$S\left({{\rm{a}}},{{\rm{b}}}\right)=\frac{{\left\langle {{\rm{a}}},{{\rm{b}}}\right\rangle }_{\Omega }}{\sqrt{{\left\langle {{\rm{a}}},{{\rm{a}}}\right\rangle }_{\Omega }\,{\left\langle {{\rm{b}}},{{\rm{b}}}\right\rangle }_{\Omega }}}$$

where the inner product is given by ${\left\langle {{\bf{a}}},{{\bf{b}}}\right\rangle }_{{{\mathbf{\Omega }}}}={{{\bf{a}}}}^{{{\boldsymbol{T}}}}\,{{\mathbf{\Omega }}}\,{{\bf{b}}}$. This formulation captures functional relationships between genes by leveraging the structured connectivity information in the PPI network.

### Clinical correlation and patient cohort analysis

For the TCGA AML cohort, RNA-seq data and corresponding clinical information were obtained from the Genomic Data Commons Data Portal. The Beat AML dataset, including RNA-seq counts and clinical annotations, was accessed through the companion data portal. The Myeloid Program gene signature was defined as the top 25% of genes based on their regulatory weights within the identified program (approximately 85 genes). The association between program activity and overall survival was assessed using Cox proportional hazards regression models, implemented in the ‘survival’ R package (v3.4-0). Hazard ratios (HR) with 95% confidence intervals and corresponding *p*-values were calculated for each program, with adjustment for known prognostic factors including age, cytogenetic risk group, and mutation status of key genes (*FLT3, NPM1, TP53, DNMT3A, CEPBA, RUNX1*). Statistical significance was determined using two-sided Wilcoxon rank-sum tests with Benjamini–Hochberg correction for multiple testing. Box plots were used to visualize the distribution of program activity scores across different patient subgroups, with significant comparisons (adjusted *p* < 0.05) indicated.

### Statistical analysis

No statistical methods were used to pre-determine sample sizes. The sample size for single-cell experiments was based on capturing a sufficient number of cells per guide to detect transcriptomic shifts and ensure statistical power for identifying differentially expressed genes. For single-cell transcriptomic comparisons, the non-parametric Wilcoxon rank-sum test was employed, which does not assume a normal distribution of the data. For continuous variables, including drug response and program activity comparisons, data were evaluated for normality and homogeneity of variance prior to testing. All pharmacological experiments were performed in at least three independent biological replicates. All statistical tests were two-sided unless otherwise noted. For differential expression, Seurat’s Wilcoxon rank-sum test was used with Benjamini–Hochberg correction for multiple comparisons. Survival analysis utilized Cox proportional hazards regression adjusted for clinical covariates. Center values in graphs represent the mean, and error bars represent the standard deviation (s.d.). *, **, ***, and **** denote *P* values < 0.05, 0.01, 0.001, and 0.0001, respectively. n.s. denotes not significant.

## Supplementary information

Supplemental materials

Supplementary Table 1

Supplementary Table 2

Supplementary Table 3

Supplementary Table 4

## Data Availability

The scRNA-seq and bulk RNA-seq data will be available at the Gene Expression Omnibus (GEO) with the accession number GSE294098 and GSE294096, respectively.

## Code availability

The scripts for next-generation sequencing data analyses are available from the corresponding authors upon request. Further information and requests for resources and reagents should be directed to Rui Lu (ruilu1@uabmc.edu) and Changde Cheng (ccheng3@uab.edu).
