# Supplementary material for: A Perturb-seq map of a differentiation hub reveals synergistic vulnerabilities in KMT2A-rearranged acute myeloid leukemia
Source: Leukemia. 2026 Mar 25;40(5):996–1008. doi: 10.1038/s41375-026-02917-2 (PMC13149302; doi:10.1038/s41375-026-02917-2)
Supplement: Supplementary file 1 — Supplemental materials [file 41375_2026_2917_MOESM1_ESM.pdf]

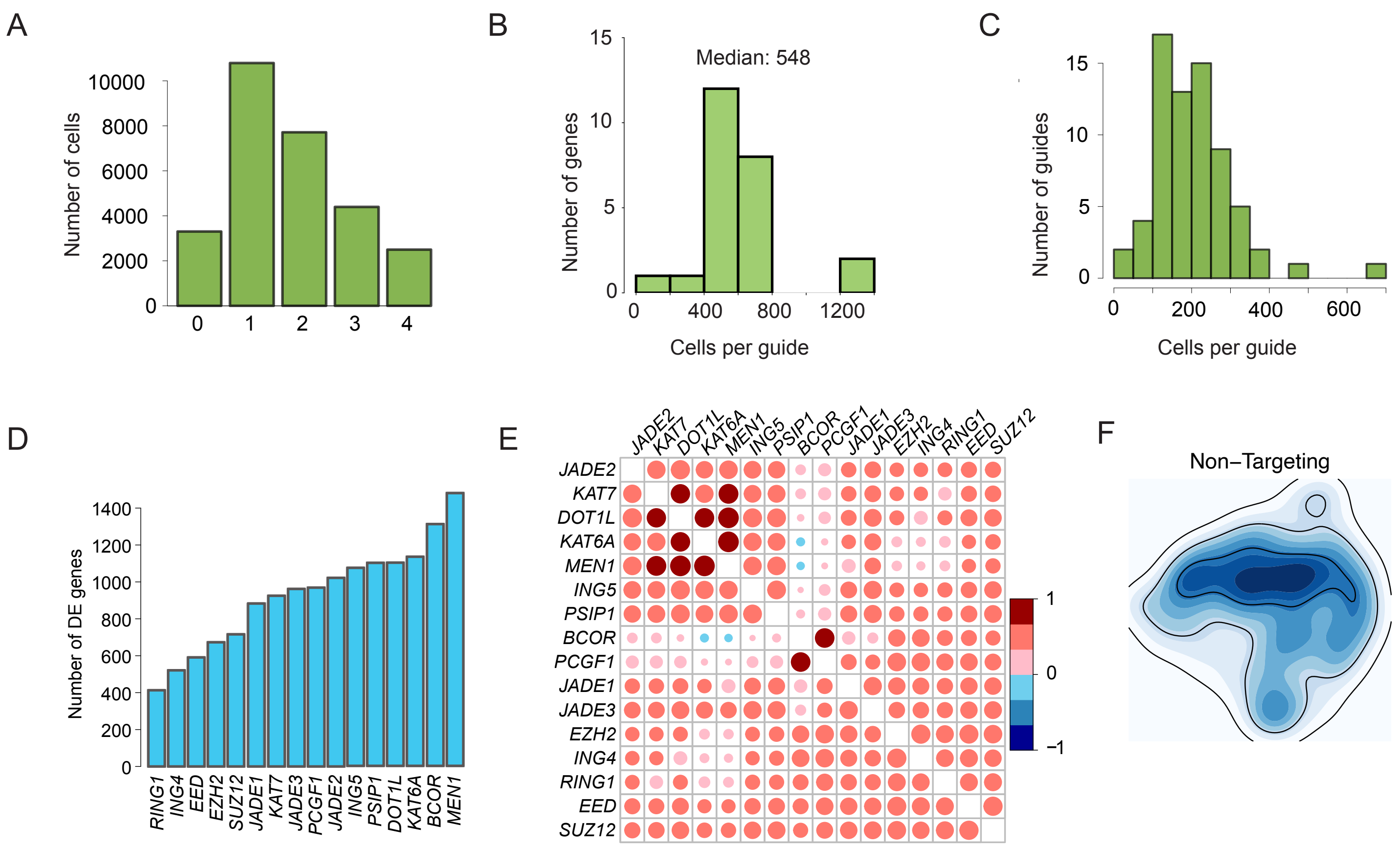

A
Number of cells
0
2000
4000
6000
8000
10000
0 1 2 3 4
B
Median: 548
Number of genes
0 5 10 15
Cells per guide
0 400 800 1200
C
Number of guides
0 5 10 15
Cells per guide
0 200 400 600
D
Number of DE genes
0 200 400 600 800 1000 1200 1400
RING1 ING4 EED EZH2 SUZ12 JADE1 KAT7 JADE3 PCGF1 JADE2 ING5 PSIP1 DOT1L KAT6A BCOR MEN1
E
JADE2 KAT7 DOT1L KAT6A MEN1 ING5 PSIP1 BCOR PCGF1 JADE1 JADE3 EZH2 ING4 RING1 EED SUZ12
1
0
−1
F
Non−Targeting

Supplementary Figure 1. High-throughput single-cell perturbation of epigenetic regulators in AML. A. Bar graph showing the distribution of cells containing 0, 1, 2, or 3 distinct guides per cell, indicating the majority of cells were successfully transduced with a single guide RNA. **B**. Cell Histogram depicting the number of cells associated with each perturbed gene, with a median of 548 cells per gene. **C**. Bar graph representing the number of cells per guide RNA and showing a median of 182 cells per guide. **D**. Bar plot showing the number of differentially expressed genes (DEGs) for each perturbed target gene. The height of each bar corresponds to the DEG count. **E**. Correlation matrix of expression changes for the perturbed genes. Positive correlations within chromatin complexes are highlighted in red. **F.** Density plot visualizing the UMAP projection of cells transduced with non-targeting control sgRNAs.

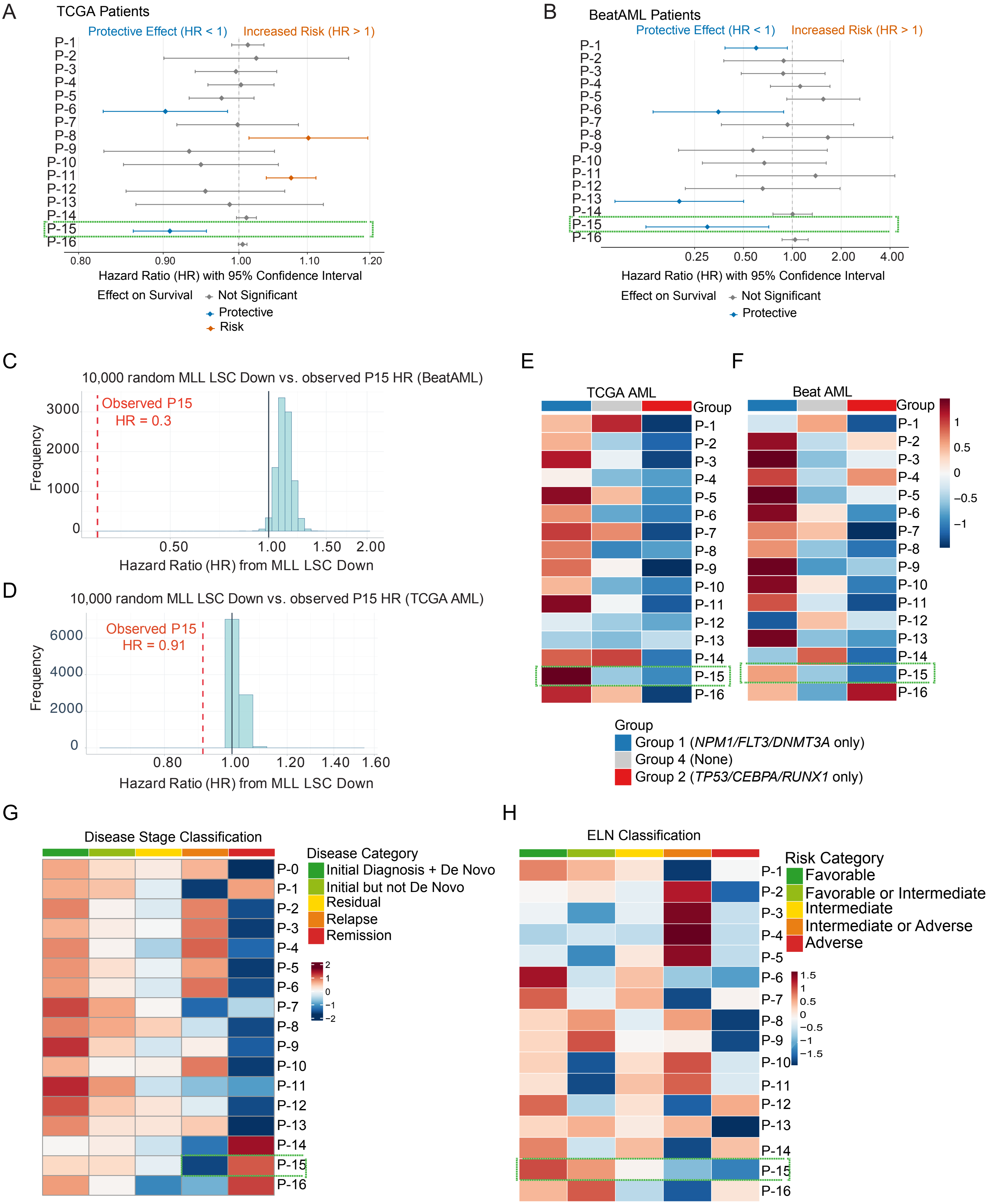

A
TCGA Patients
Protective Effect (HR < 1)
Increased Risk (HR > 1)
P-1
P-2
P-3
P-4
P-5
P-6
P-7
P-8
P-9
P-10
P-11
P-12
P-13
P-14
P-15
P-16
0.80
0.90
1.00
1.10
1.20
Hazard Ratio (HR) with 95% Confidence Interval
Effect on Survival
Not Significant
Protective
Risk
B
BeatAML Patients
Protective Effect (HR < 1)
Increased Risk (HR > 1)
0.25
0.50
1.00
2.00
4.00
Hazard Ratio (HR) with 95% Confidence Interval
Effect on Survival
Not Significant
Protective
C
10,000 random MLL LSC Down vs. observed P15 HR (BeatAML)
Observed P15
HR = 0.3
Frequency
Hazard Ratio (HR) from MLL LSC Down
D
10,000 random MLL LSC Down vs. observed P15 HR (TCGA AML)
Observed P15
HR = 0.91
Frequency
Hazard Ratio (HR) from MLL LSC Down
E
TCGA AML
Group
F
Beat AML
Group
Group 1 (NPM1/FLT3/DNMT3A only)
Group 4 (None)
Group 2 (TP53/CEBPA/RUNX1 only)
G
Disease Stage Classification
Disease Category
Initial Diagnosis + De Novo
Initial but not De Novo
Residual
Relapse
Remission
P-0
H
ELN Classification
Risk Category
Favorable
Favorable or Intermediate
Intermediate
Intermediate or Adverse
Adverse

Supplementary Figure 2. Computational modeling identified a clinically relevant myeloid differentiation program. A-B. Forest plots showing the prognostic association (Hazard Ratio for overall survival) of all 17 gene programs in the TCGA AML cohort **(A)** and Beat AML cohort **(B)**. Permutation analysis of the prognostic specificity of P-15. **C-D**. The observed Hazard Ratio of the Myeloid Program (red dashed line) was compared against a null distribution of HRs generated from 10,000 random gene signatures derived from the MLL LSC-down gene set in the Beat AML **(C)** and TCGA **(D)** cohorts. **E.** Heatmap showing significantly higher Myeloid Program (P-15) expression in NPM1-mutated AML and lower Myeloid Program expression in TP53-mutated AML in TCGA AML cohort. **F.** Heatmap showing significantly higher Myeloid Program (P-15) expression in NPM1-mutated AML and lower Myeloid Program expression in TP53-mutated AML in Beat AML cohort. **G.** Heatmap showing significant enrichment of the Myeloid Program in favorable-risk AML patients compared to adverse-risk patients according to ELN 2017 classification. **H.** Heatmap showing significantly higher Myeloid Program expression in remission-stage patients compared to those with relapsed or residual disease in the Beat AML cohort.

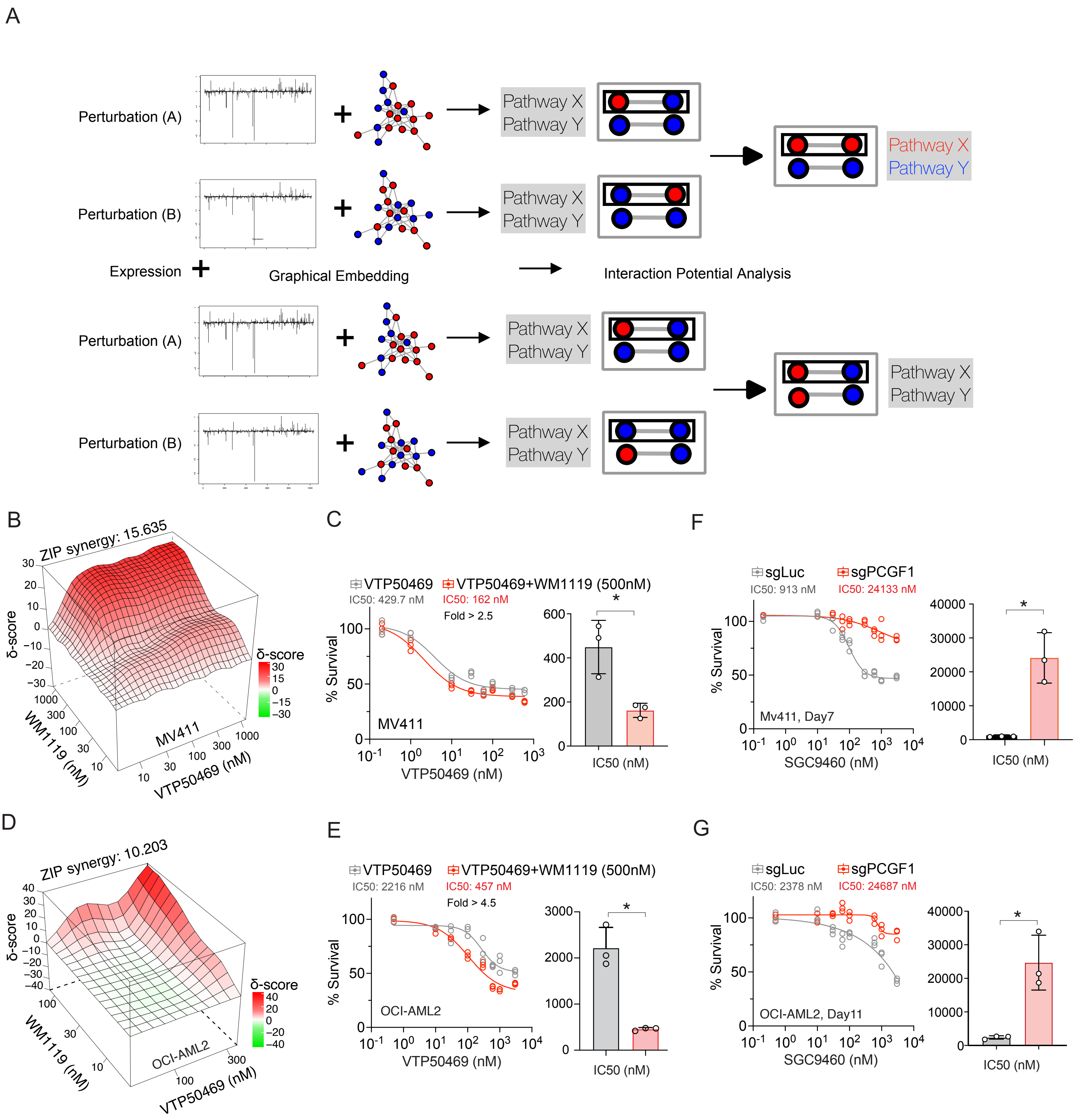
A
Perturbation (A)
Perturbation (B)
Expression
Graphical Embedding
Interaction Potential Analysis
Pathway X
Pathway Y
B
ZIP synergy: 15.635
δ-score
MV411
WM1119 (nM)
VTP50469 (nM)
C
VTP50469
IC50: 429.7 nM
VTP50469+WM1119 (500nM)
IC50: 162 nM
Fold > 2.5
% Survival
MV411
VTP50469 (nM)
IC50 (nM)
D
ZIP synergy: 10.203
OCI-AML2
E
VTP50469
IC50: 2216 nM
VTP50469+WM1119 (500nM)
IC50: 457 nM
Fold > 4.5
OCI-AML2
F
sgLuc
IC50: 913 nM
sgPCGF1
IC50: 24133 nM
Mv411, Day7
SGC9460 (nM)
G
sgLuc
IC50: 2378 nM
sgPCGF1
IC50: 24687 nM
OCI-AML2, Day11

Supplementary Figure 3. Targeting the MEN1/DOT1L-KAT6A hub reveals a potent synergistic vulnerability in AML. A. Description of the methodology used to calculate weighted interaction indices for synergy and antagonism, based on the differential expression of gene sets related to cell cycle, DNA damage response, and apoptosis following single and dual perturbations. **B.** 3D ZIP synergy heatmap showing the interaction between the Menin inhibitor VTP50469 and the KAT6A inhibitor WM1119 in MV4-11 cells. **C.** Dose-response curve and corresponding IC50 comparison for VTP50469 alone versus in combination with WM1119 (500 nM) in MV4-11 cells. **D.** 3D ZIP synergy heatmap showing the interaction between VTP50469 and WM1119 in OCI-AML2 cells. **E.** Dose-response curve and corresponding IC50 comparison for VTP50469 alone versus in combination with WM1119 (500 nM) in OCI-AML2 cells. **F-G.** Dose-response curves for the DOT1L inhibitor SGC0946 in **(F)** MV4-11 and **(G)** OCI-AML2 cells transduced with control (sgLuc) or PCGF1-targeting (sgPCGF1) sgRNAs.

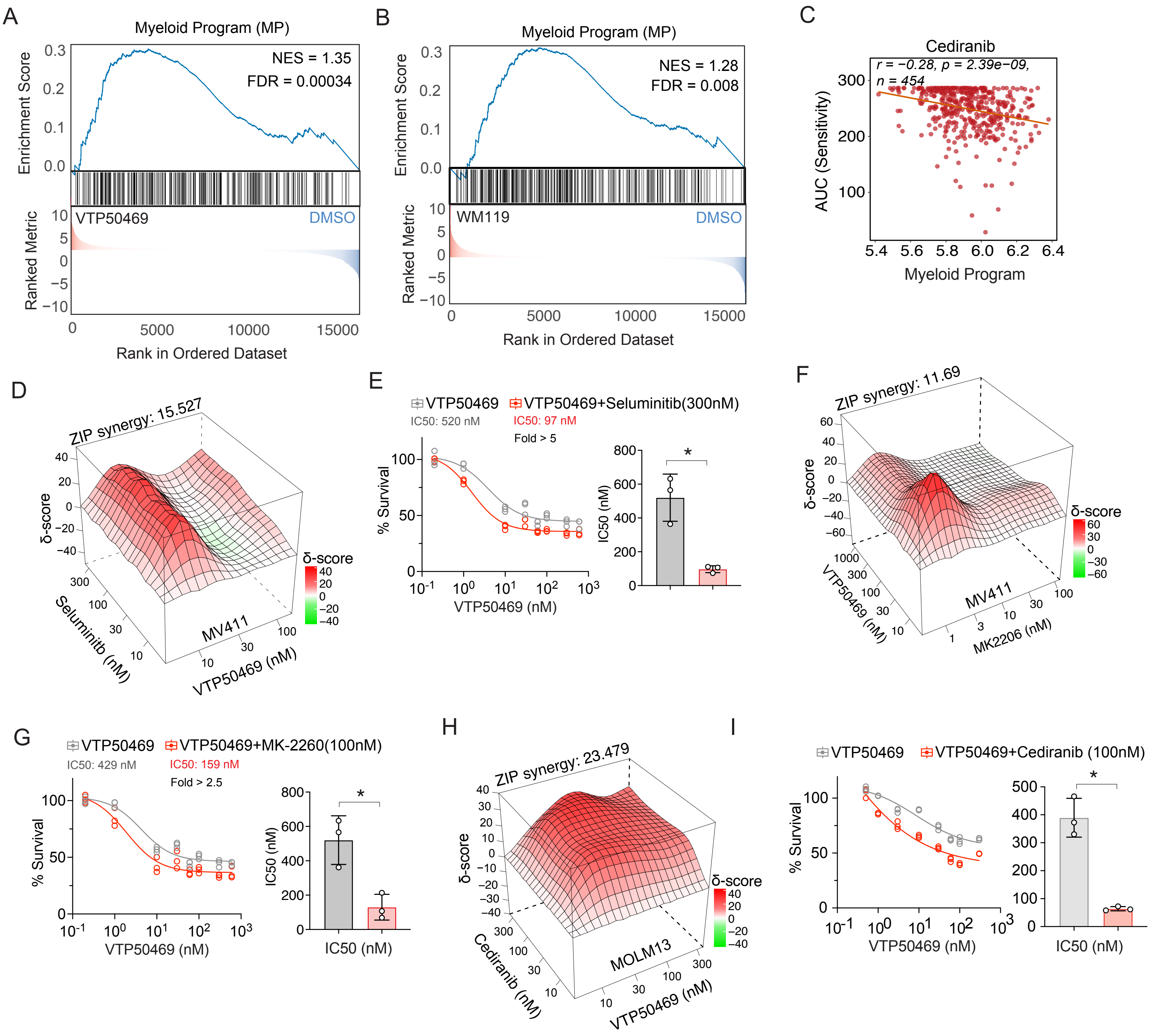
A
Myeloid Program (MP)
NES = 1.35
FDR = 0.00034
Enrichment Score
Ranked Metric
VTP50469
DMSO
Rank in Ordered Dataset
B
Myeloid Program (MP)
NES = 1.28
FDR = 0.008
WM119
DMSO
Rank in Ordered Dataset
C
Cediranib
r = −0.28, p = 2.39e−09, n = 454
AUC (Sensitivity)
Myeloid Program
D
ZIP synergy: 15.527
δ-score
MV411
Seluminitib (nM)
VTP50469 (nM)
E
VTP50469
VTP50469+Seluminitib(300nM)
IC50: 520 nM
IC50: 97 nM
Fold > 5
% Survival
VTP50469 (nM)
IC50 (nM)
F
ZIP synergy: 11.69
δ-score
MV411
VTP50469 (nM)
MK2206 (nM)
G
VTP50469
VTP50469+MK-2260(100nM)
IC50: 429 nM
IC50: 159 nM
Fold > 2.5
% Survival
VTP50469 (nM)
IC50 (nM)
H
ZIP synergy: 23.479
δ-score
MOLM13
Cediranib (nM)
VTP50469 (nM)
I
VTP50469
VTP50469+Cediranib (100nM)
% Survival
VTP50469 (nM)
IC50 (nM)

Supplementary Figure 4. The Myeloid Program score predicts drug sensitivity and identifies novel synergies with Menin inhibition. A. GSEA plots showing the enrichment of the Myeloid Program in MOLM-13 cells treated with Menin inhibitor VTP50469 alone. **B**. GSEA plots showing the enrichment of the Myeloid Program in MOLM-13 cells treated with KAT6A inhibitor WM1119 alone. **C**. Scatter plot showing the negative correlation between Myeloid Program activity and sensitivity to the VEGFR inhibitor Cediranib in the Beat AML cohort. **D.** ZIP synergy score for combination of VTP50469 and Selumetinib in MV4-11 cells. **E.** Dose-response curves showing a reduction in the VTP50469 IC50 when combined with Selumetinib. **F.** ZIP synergy score for combination of VTP50469 with MK-2206 in MV4-11 cells. **G.** Dose-response curves showing a reduction of IC50 of VTP50469 when combined with MK-2206. **H**. ZIP synergy score for the combination of VTP50469 and Cediranib. **I**. Dose-response curves showing a significant reduction in the IC50 of VTP50469 when combined with Cediranib.

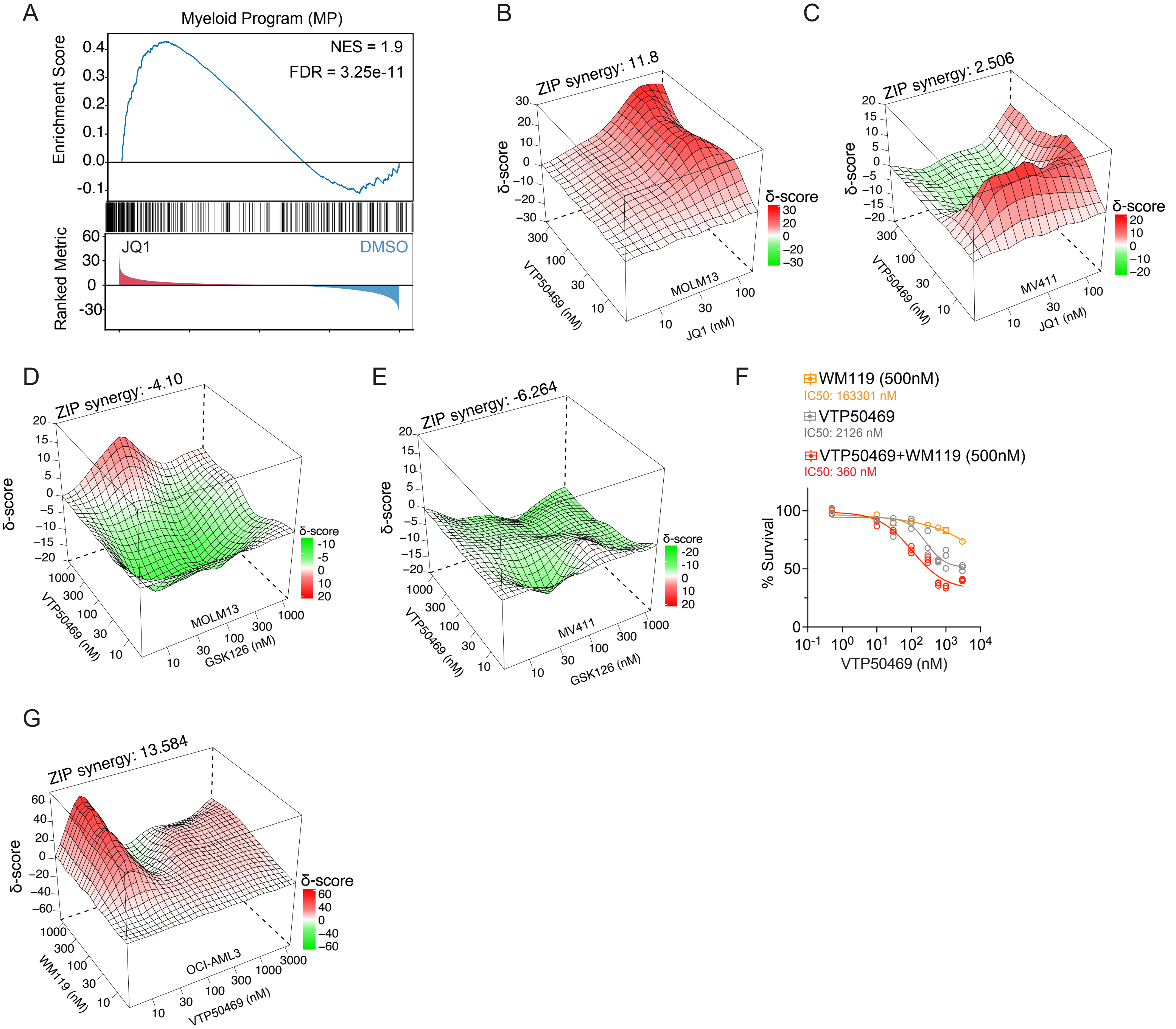
A
Myeloid Program (MP)
NES = 1.9
FDR = 3.25e-11
Enrichment Score
Ranked Metric
JQ1
DMSO
B
ZIP synergy: 11.8
δ-score
MOLM13
VTP50469 (nM)
JQ1 (nM)
C
ZIP synergy: 2.506
δ-score
MV411
VTP50469 (nM)
JQ1 (nM)
D
ZIP synergy: -4.10
δ-score
MOLM13
VTP50469 (nM)
GSK126 (nM)
E
ZIP synergy: -6.264
δ-score
MV411
VTP50469 (nM)
GSK126 (nM)
F
WM119 (500nM)
IC50: 163301 nM
VTP50469
IC50: 2126 nM
VTP50469+WM119 (500nM)
IC50: 360 nM
% Survival
VTP50469 (nM)
G
ZIP synergy: 13.584
δ-score
OCI-AML3
WM119 (nM)
VTP50469 (nM)

Supplementary Figure 5. Specificity of the Myeloid Program. A. Gene Set Enrichment Analysis (GSEA) showing that pharmacological inhibition of BRD4 (JQ1) in THP-1 cells significantly activates the Myeloid Program (NES = 1.91, FDR = 3.25e-11). **B-C.** ZIP Synergy heatmaps for the combination of the Menin inhibitor VTP50469 and the BRD4 inhibitor JQ1 in **(B)** MOLM-13 cells and **(C)** MV4-11 cells. **D-E.** ZIP synergy heatmaps for VTP50469 combined with the EZH2 inhibitor GSK126 in **(D)** MOLM13 and **(E)** MV4-11. **F.** Dose-response curve and IC50 comparison showing that WM1119 sensitizes the VTP50469. **G.** ZIP synergy scores showing synergy between VTP50469 and the KAT6A inhibitor WM1119 in OCI-AML3 cell line.

Supplementary Table Legends:

Supplementary Table 1. List of the 16 key epigenetic regulators targeted in the Perturb-seq screen and the three sgRNA sequences designed for each target gene.

Supplementary Table 2. Differentially expressed genes for each of the 16 perturbations versus control cells in the Perturb-seq dataset.

Supplementary Table 3. Complete gene lists for the 17 transcriptional programs (P-0 to P-16) identified by regularized linear modeling of the Perturb-seq data.

Supplementary Table 4. Functional enrichment analysis (gene ontology biological process and KEGG pathways) of the 17 transcriptional programs. Hypergeometric test with Benjamini–Hochberg FDR correction.
